# Ten Simple Rules to Achieve Conference Speaker Gender Balance

**DOI:** 10.1371/journal.pcbi.1003903

**Published:** 2014-11-20

**Authors:** Jennifer L. Martin

**Affiliations:** Institute for Molecular Bioscience, University of Queensland, Brisbane, Australia; National Institutes of Health, United States of America

Recently, the quantum molecular science world was in uproar [1], [2]. The preliminary list of approximately 25 speakers for the International Congress of Quantum Chemistry (ICQC) was published online, with no women speakers listed. One reaction to this list was to set up a petition to “condemn gender-biased discriminatory practices of which ICQC-2015 is the most recent example” [3]. This resulted in an apology and a new speaker list with six women speakers [4].

Sadly though, this is not an isolated incident: men-only invited conference speaker lists are all too common [5].

How can we get gender balance right? To begin with, it's worth reminding ourselves why gender balance is important.

First, it's critical for the future of science that young women and men can see real evidence that scientists can succeed regardless of gender. So, if we are going to encourage women into careers in science we need also to provide role models for them to aspire to. We need to show that being a woman and being a successful scientist are not mutually exclusive. One way of doing that is to give women scientists a platform to present their research. If we don't address gender balance in speaker programs, we will continue to normalise a gendered stereotype of scientific leadership. Then when crunch time comes, women will continue to leave in far greater numbers than men [6]–[9] in part because they see no path ahead for themselves. And that means scientific research potentially loses half of its brightest talent.

Moreover, a speaking invitation contributes enormously to the profile of a researcher. By extending more invitations to women and other under-represented sections of the academic community, we provide a boost to their visibility and their track record. This will help them to progress by raising their national and international profile and help support their applications for grants, academic positions, and fellowships.

Finally, conferences and symposia are great ways of generating new collaborations, new ideas, and new directions in science. If we keep inviting the same people, and the same types of people, over and over again, we limit the diversity of thought and, potentially, the opportunities for innovation.

So, here are ten simple rules to achieve conference speaker gender balance.

## Rule 1: Collect the Data

Count the number of women and men attending a conference, or the number of women and men who have membership of a professional society, or the number of women and men who are employed or studying at a University department. If the same conference/seminar series has been running for a number of years, averaged data could be used (over the past five years, for example). When running a conference for the first time or collecting information about society membership, make sure to include gender as one of the questions to allow this base rate to be generated. Use the information to determine the gender balance of the conference, seminar series, or department. Of course, this may change over time, so it's worth checking every few years.

## Rule 2: Develop a Speaker Policy

A speaker policy captures what the committee is trying to achieve for its members and audience when putting together the speaker program. It can also help the committee measure outcome. A policy may state, for example, that the conference committee wants to achieve a gender balance of speakers that roughly reflects that of its audience. Depending on the conference or meeting, the policy might include scientific diversity, geographical distribution, ethnicity, and level of seniority in the speaker policy. If you are not sure what a conference policy looks like, check examples written by others, such as the Lorne Proteins conference [10] or the Crystal29 conference [11]. The policy can be quite simple and, yet, still effective. Data from Rule 1 above will feed into and perhaps help modify the policy, but a policy should be developed immediately. An anti-harassment statement should also be included in the conference policy [12].

## Rule 3: Make the Policy Visible

It's no use having a policy if no one knows about it. Make it visible. Put it online for everyone to see. Make a direct link to it on the conference or symposia website and put it on your Facebook page. Provide it to the organising committee, the program committee, the society executive, and the departmental research committee. Send it to the chairs of the sessions, send it to the invited speakers. Make sure everyone knows right from the start that the conference committee is serious about getting gender balance right. Don't make gender balance an afterthought.

## Rule 4: Establish a Balanced and Informed Program Committee

If the conference program committee is not diverse, then neither will be the speaker list. When I've asked male members of conference organising committees, or men in the audience, about poor gender balance or even good gender balance, they invariably tell me they don't notice the gender balance one way or the other. So to avoid the potential issue of gender blindness, make sure that those inviting and selecting the speakers (the program committee or symposia chairs) are familiar with the conference policy and that the program committee itself is diverse, informed, and gender balanced [13].

## Rule 5: Report the Data

The next step is to see how well the conference, speaker series, or symposium meets its stated policy goals. To do this, calculate the percentage of women in the list of invited speakers. And do the same for the selected speakers. How do these numbers compare with the percentage of women in the audience? If the percentage of women speakers is consistently lower than that of the audience, then maybe it's time to overhaul the policy. Maybe the gender balance of the program committee needs to be changed. In any case, report the numbers on the website, on the same page as the policy. Comment on the data. For example, “Our stated policy is to achieve a gender balance of >40% women in our speaker list (50% of our delegates are women). Overall, 35% of our speakers were women. This is good, but we can do better.” Ask for feedback.

To go one step further, you could establish a website to “crowdsource and collate the gender breakdown of conferences” [14] to identify and promote conferences that best support gender balance.

## Rule 6: Build and Use Databases

Some people find it difficult to come up with names of women speakers, compared with men speakers. Some say there aren't enough senior or mid-career women in the field to get a balanced program. When I got this response last year after querying a proposed speaker list, I arranged to meet with the organiser to brainstorm a new list. We collected enough names in one afternoon to fill two or three conferences. The list of potential women could include younger, up-and-coming women who would benefit from the exposure.

It's also worthwhile looking through lists of women scientists that have been compiled to help conference organisers—see, for example [15]–[17]. This is by no means an exhaustive list of sites, so please add more. And if you can't find a list in your field, consider compiling one yourself.

## Rule 7: Respond to Resistance

Expect to meet resistance. Most criticisms are easily addressed by establishing a dialogue with those who are critical about establishing a policy, and you can prepare in advance.

Some will say the most important thing is not diversity or the number of women speakers, the most important thing is having a high-quality program. “We only select the best speakers.” Addressing gender balance is not inconsistent with a high-quality program. Perhaps point them to the implicit association site [18].

Similarly, some will say the most important thing is diversity of thought, not speaker diversity. Diversity in life experience equals diversity of thought. Again, having a gender-balanced program is not inconsistent with diversity of thought. On the flip side, inviting the same people over and over again does not address diversity of thought.

Some will say, yes let's have a policy, but let's not make it public because that makes it look like we've had a problem in the past and are now apologising for it. There is no point having a policy if no one knows about it. Put it online. See Rule 3.

Some will say that a policy isn't needed because gender balance is achieved already. Check the data. See Rule 1. Maybe gender balance is OK, but it's important to ensure that invisible inequities do not prevail.

## Rule 8: Support Women at Meetings

Women often have primary caring responsibility for children. This can limit their ability to travel and to attend conferences. Professor Jonathan Eisen (UCD) has stated: “If you're going to spend money on an open bar instead of childcare…you should rethink what you're doing” [19]. Some universities are now offering travel support for partners or nannies to attendees who would otherwise not be able to accept conference speaking invitations. Perhaps conferences could do the same. Ideas on why women don't accept invitations and how to support their attendance, such as providing a childcare center and avoiding gendered language, have been outlined [20].

## Rule 9: Be Family-Friendly

In those cases where the conference is large enough, and the number of attendees bringing children is significant, it may be possible to provide a family room. This allows delegates with children to watch conference presentations via video link. Also consider carefully the social events to be scheduled at your conference. Make sure these are appropriate.

## Rule 10: Take the Pledge

Finally, the most important and powerful step of all. When you are invited to help organise, attend, or speak at a conference, ask to see the conference speaker policy before you accept. If there isn't one, which is usually the case, offer to help draft one. You could also ask to see the list of invited speakers and if there isn't a reasonable gender balance, just say no. That's what a group of Scandinavian men have pledged: to say no thanks, when there are no/few women speakers [21].

You could also sign the online petition set up by Virginia Valian and Dan Sperber [22] in which “signatories commit to accepting talk invitations only from conferences that have made good-faith efforts to include women.”

So, those are the ten simple rules.

One day, hopefully not too far away, I'd like to think we won't actually need conference speaker policies anymore. The process of selecting and supporting a broad, diverse, balanced list of high-quality speakers will be as automatic as flicking to the next slide on a PowerPoint presentation.
